# Supplementary material for: The causal role of circulating inflammatory markers in osteoporosis: a bidirectional Mendelian randomized study
Source: Front Immunol. 2024 Jul 18;15:1412298. doi: 10.3389/fimmu.2024.1412298 (PMC11291241; doi:10.3389/fimmu.2024.1412298)
Supplement: Supplementary file 1 [file DataSheet_1.zip › supplementary material/OSTEOPOROSIS-CIM/GCST90274828.tsv.gz_forest.pdf]

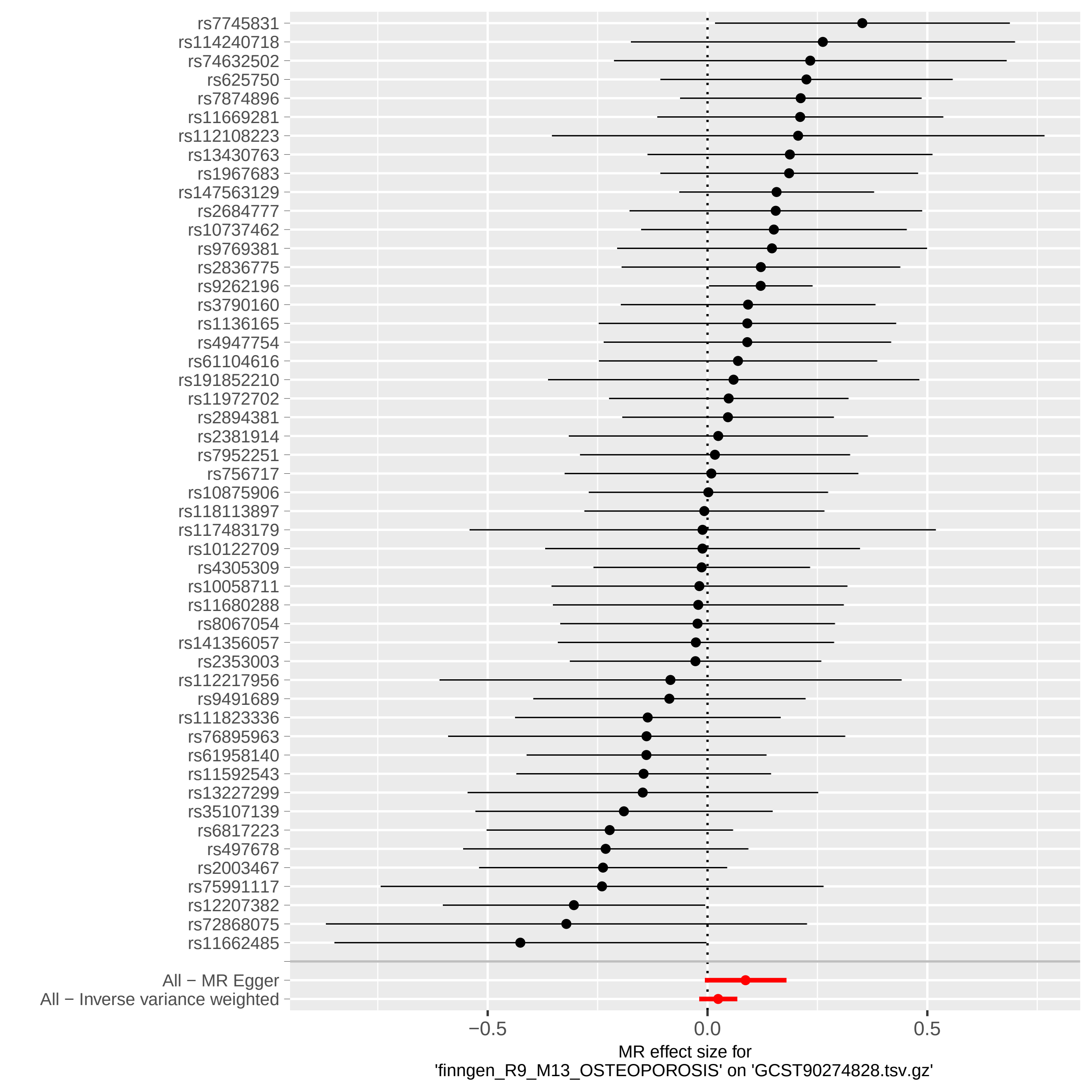
rs7745831
rs114240718
rs74632502
rs625750
rs7874896
rs11669281
rs112108223
rs13430763
rs1967683
rs147563129
rs2684777
rs10737462
rs9769381
rs2836775
rs9262196
rs3790160
rs1136165
rs4947754
rs61104616
rs191852210
rs11972702
rs2894381
rs2381914
rs7952251
rs756717
rs10875906
rs118113897
rs117483179
rs10122709
rs4305309
rs10058711
rs11680288
rs8067054
rs141356057
rs2353003
rs112217956
rs9491689
rs111823336
rs76895963
rs61958140
rs11592543
rs13227299
rs35107139
rs6817223
rs497678
rs2003467
rs75991117
rs12207382
rs72868075
rs11662485
All − MR Egger
All − Inverse variance weighted
−0.5
0.0
0.5
MR effect size for
'finngen_R9_M13_OSTEOPOROSIS' on 'GCST90274828.tsv.gz'
